# Rotational Ankle Fracture Dislocation With Associated Lisfranc Fracture

**DOI:** 10.7759/cureus.17148

**Published:** 2021-08-13

**Authors:** Teigen Goodeill, Jason Lin, Jacqueline Krumrey

**Affiliations:** 1 Orthopedic Surgery, Good Samaritan Regional Medical Center, Corvallis, USA

**Keywords:** lisfranc fracture, intercuneiform, bimalleolar, ankle fracture, open reduction internal fixation, missed injury

## Abstract

While ankle fractures most often result from a rotational injury, Lisfranc injuries are more commonly associated with an axial load on a plantarflexed foot. Due to differing mechanisms of injury, rotational ankle fractures with Lisfranc injuries are uncommon and rarely discussed in the literature. Here we present a case of a rotational ankle fracture-dislocation with a concomitant Lisfranc injury. The Lisfranc injury, which was ultimately treated nonoperatively, was discovered seven weeks after operative fixation of the ankle fracture. At the last follow-up nine months after the initial injury, the patient had mild midfoot soreness with activity but no evidence of deformity or arch collapse. Although no deformity was observed in our patient, missed Lisfranc injuries may result in significant functional deficits indicating the importance of recognizing the possibility of a dual injury.

## Introduction

Whereas ankle fractures are quite common, a Lisfranc injury is rarer [1] and is often missed or diagnosed late [2]. The Lisfranc injury, which is described as an intra-articular injury at the tarsometatarsal joint, is estimated to make up 0.2% of all fractures [1]. As they are less common, Lisfranc injuries have a propensity to be overlooked in the setting of multiple traumas and can be missed in up to 20% of cases [3]. A missed or late Lisfranc injury diagnosis may lead to worsened outcomes as it may be too late to achieve reduction [4]. This may result in activity limitations, flatfoot deformity, and loss of the longitudinal arch of the foot [5].

The typical mechanism leading to a Lisfranc injury is an axial load on a plantarflexed foot and, less commonly, a twisting mechanism causing forced forefoot abduction [6]. Lisfranc injuries are associated with impaired function and gait when compared to healthy subjects [7]. While an axial load is the most common force leading to a Lisfranc injury, it is generally a rotational mechanism that results in ankle fractures [8]. The Lauge-Hansen classification, a commonly used ankle fracture classification system, attempts to describe specific fracture patterns by describing the position of the foot at the time of the traumatic event and the deforming force on the ankle. Supination external rotation injuries make up 40-70% of ankle fractures [9]. Understanding the mechanisms leading to ankle fractures allows surgeons to better characterize ankle fractures and obtain and maintain reduction [10].

We report the case of a rotational bimalleolar ankle fracture-dislocation with a concomitant Lisfranc fracture. Because of the difference in mechanism and energy transfer between Lisfranc and ankle fracture-dislocation injuries, an ankle fracture-dislocation with an associated Lisfranc injury is uncommon. Only one other case was discovered upon literature review [11]. We aim to emphasize the importance of performing a complete foot and ankle examination during the initial trauma evaluation.

## Case presentation

**Figure 1:**
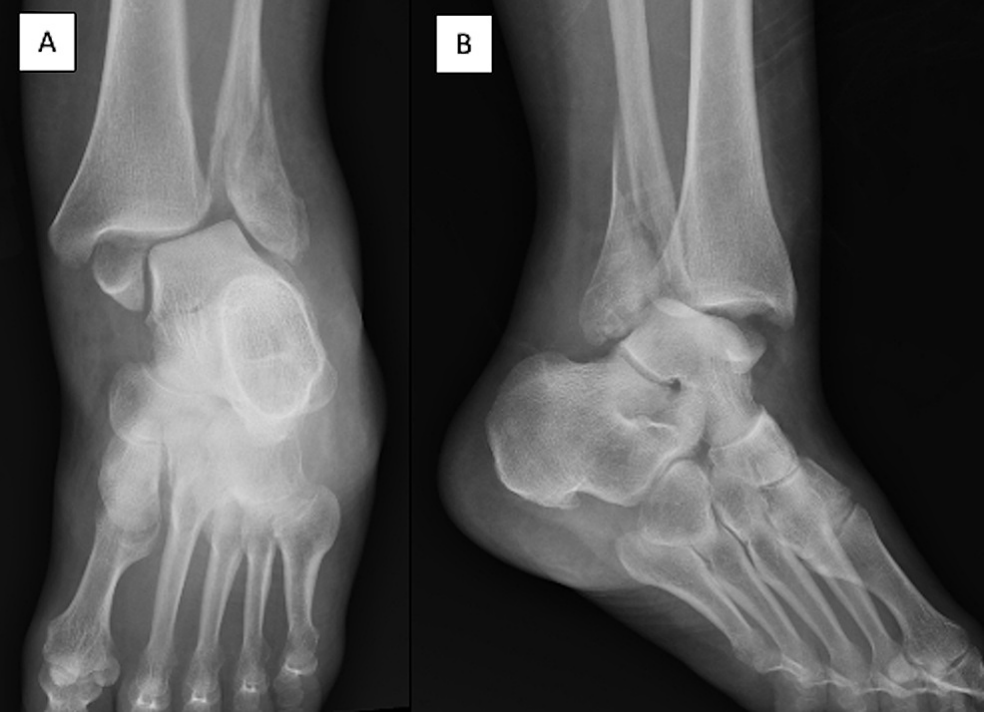
Index injury radiographs. AP (A) and lateral (B) radiographs of the right ankle demonstrating a bimalleolar ankle fracture-dislocation. AP = anteroposterior

A 53-year-old female with a past medical history of Hashimoto’s disease, obesity, and adjustment disorder slipped when stepping out of the shower resulting in pain, obvious deformity of the left ankle, and an inability to bear weight. She presented to the emergency department (ED) where she was found to have an intact neurovascular examination to the foot, obvious ankle deformity, and no skin breakdown. Initial anteroposterior and lateral radiographs obtained by the ED physician demonstrated a left bimalleolar ankle fracture-dislocation. Radiographs (Figure 1) demonstrated a transverse medial malleolus fracture, an obliquely oriented lateral malleolus fracture extending to the syndesmosis, lateral talar subluxation, and posterior talar dislocation. This injury pattern is consistent with a supination and external rotation injury.

The ankle fracture was reduced and splinted by the orthopedic trauma team under procedural sedation, and the patient was instructed to remain nonweight bearing (NWB) and follow up within the next week in the orthopedic trauma clinic. The day after the initial ED consult, the patient returned to the ED due to poor pain management and concern for compartment syndrome. An evaluation was completed by the orthopedic team without concern for compartment syndrome, but the patient did receive a nerve block by the on-call anesthesiologist to improve pain control. Outpatient follow-up in five days, which is within the standard timeline for ankle fracture follow-up at this clinic, noted that the patient’s pain level was tolerable, and she was scheduled for open reduction internal fixation (ORIF) in another four days.

**Figure 2:**
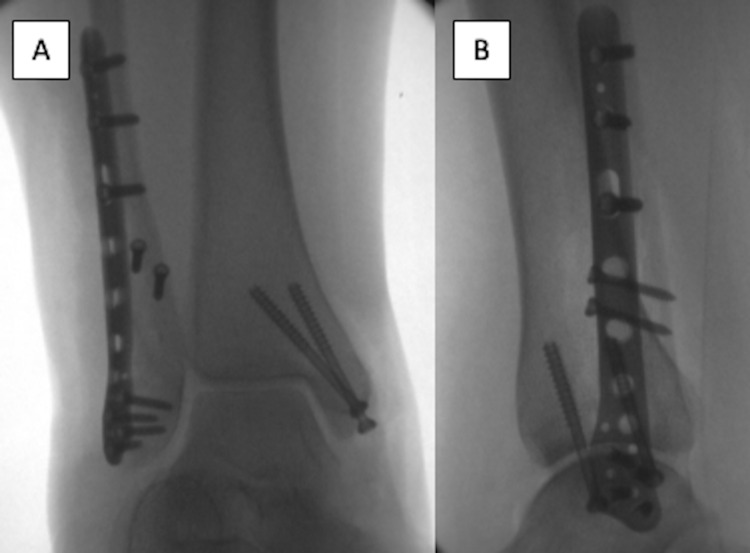
Bimalleolar ankle fracture surgical fixation. Intraoperative images of the ankle ORIF fixation using a lag screw and bridge plating technique for the lateral malleolus and two partially threaded lag screws for medial malleolus fixation. ORIF = open reduction internal fixation

Nine days after the injury, left ankle ORIF (Figure 2) was performed with a lag screw and neutralization plate for the lateral malleolus and two cannulated partially threaded screws for fixation of the medial malleolus. An external rotation stress radiograph did not demonstrate the instability of the syndesmosis. The patient was then placed in a Robert Jones splint and instructed to remain NWB and follow up in two weeks for a wound check.

**Figure 3:**
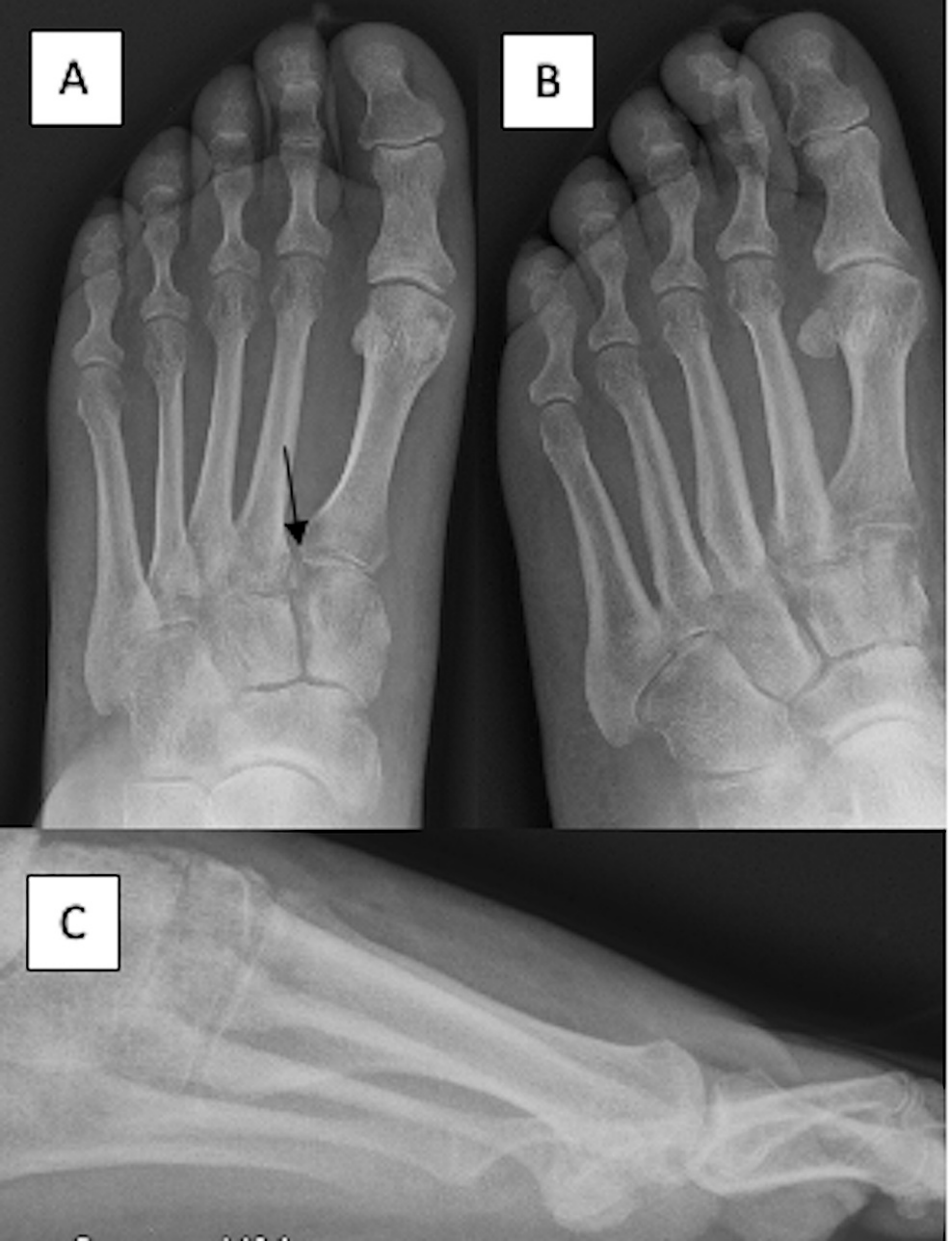
Foot radiographs. Radiographs of the foot seven weeks after the index injury demonstrating an avulsion fracture from the base of the second metatarsal consistent with Lisfranc injury.

The patient was ultimately admitted for one night on the day of the surgery due to poor pain control. At the two-week postoperative check, the patient noted that her pain was improving, the wounds were appropriately healing, and she was transitioned into a removable fracture boot. She was instructed to continue to remain NWB at the time. At the seven-week postoperative appointment, the patient had returned to working four hours per day and had minimal ankle pain. Importantly, persistent plantar ecchymosis was appreciated during this seven-week appointment. Therefore, left foot X-rays (Figure 3) were obtained which demonstrated a Lisfranc fracture avulsion at the base of the second metatarsal and 2 mm dorsal displacement of the first metatarsal base in relation to the medial cuneiform.

**Figure 4:**
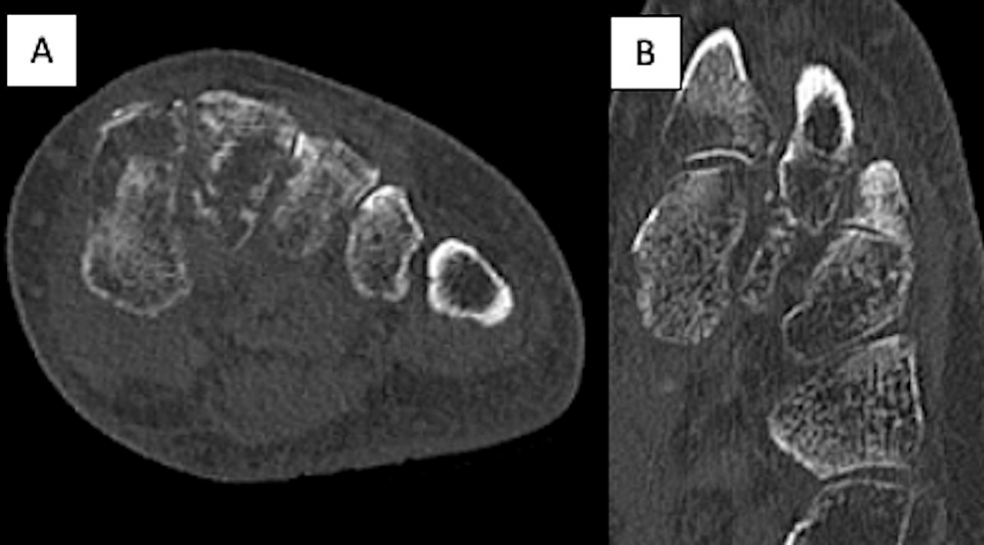
Left foot CT. Images of the left foot demonstrating a subacute Lisfranc injury with a nondisplaced avulsion fracture to the base of the second metatarsal. CT = computed tomography

At this juncture, the patient was kept NWB until a computed tomography (CT) scan (Figure 4) was obtained which demonstrated osteopenic bone, a subacute-appearing comminuted fracture at the base of the second metatarsal, a nondisplaced intra-articular medial cuneiform fracture, and no evidence of intercuneiform or metatarsal-tarsal joint subluxation/dislocation. Nonoperative management of the Lisfranc injury was recommended at this time and she was instructed to remain NWB.

Radiographs obtained at the 10-week clinic visit demonstrated signs of appropriate healing in both the ankle and foot fractures. She was allowed to bear weight as tolerated, released to work with no restrictions while remaining in her boot, and referred to physical therapy. At 15 weeks postoperatively, the patient was still using crutches for long distances due to continued left foot pain. She was prescribed a semi-rigid orthotic with arch support. At the final follow-up nine months after the initial injury, the patient noted substantial midfoot pain relief with the custom orthotics and some soreness in the middle of the foot when performing yard work or activities out of the orthotics. Physical examination showed mild hyperemia and swelling but overall physiologic hindfoot alignment with well-maintained and symmetric arches. Standing weight-bearing radiographs demonstrated mild step-off of 2 mm at the second tarsometatarsal joint with calcification along the Lisfranc ligament and subtle diastases between the second metatarsal base and the medial cuneiform/first metatarsal. Given the physical examination and radiographic findings, the Lisfranc injury was deemed stable, and she was allowed to follow up on an as-needed basis.

## Discussion

Lisfranc injuries most commonly occur from an axial loading mechanism on a plantarflexed foot [6] while ankle fractures are commonly rotational injuries [8]. Lisfranc injuries are often missed or diagnosed late [2,12,13], especially in the polytrauma patient setting [14]. A literature review of this unique injury pattern provided only one other case report of a higher energy motor vehicle accident which is more commonly associated with an axial load Lisfranc injury [11]. As this dual injury is not commonly encountered, it is important to recognize given the implications of a missed Lisfranc injury diagnosis [6]. In a study evaluating the results of a group of 55 Lisfranc injuries with a mean follow-up of 4.2 years, the authors noted posttraumatic arthritic changes in essentially all patients; however, there was a low level of correlation between the number of degenerative changes and the clinical result [4]. Additionally, a Lisfranc injury review article by Sybold and Coetzee found that 40-94% of Lisfranc injuries lead to degenerative arthritic changes despite ORIF [15].

While Lisfranc injuries are commonly thought to be associated with high-energy trauma, a recent yearlong survey performed in Norway evaluating the injury mechanism of Lisfranc injuries in 84 patients demonstrated that the most common mechanism of injury was in fact low-energy mechanisms such as fall from own height or a rotational injury to the foot [13]. Furthermore, a study performed at a military tertiary center found that in a five-year time period, 60% of operatively treated Lisfranc injuries were low-energy mechanisms [12]. Low-energy mechanisms were defined as athletic activity, ground-level twisting, fall from less than 4 feet, falling downstairs, or bike accidents [12,13]. These studies are important as they demonstrate that Lisfranc injuries may be more common in low-energy mechanisms than previously thought and that clinicians should have a higher suspicion of Lisfranc injury in low-energy trauma patients. This highlights the importance of evaluating a trauma patient. An organized, full-body and extremity, systematic evaluation is required to thoroughly assess a trauma patient [16]. It is key to avoid honing in on obvious injuries to decrease the likelihood of missing more subtle pathology [16]. We suspect that, when falling out of the shower, the patient likely planted her foot down for balance leading to the Lisfranc injury, and then upon not achieving balance, she suffered a rotational and inversion injury resulting in the ankle fracture. Although our case did not result in functional deficit or deformity nine months after ORIF of the bimalleolar ankle fracture and nonoperative management of the Lisfranc injury, the importance of recognizing the mechanism of injury and performing a complete physical examination is evident.

## Conclusions

At the final follow-up, the patient was approximately nine months post-injury and was deemed appropriate to follow up on an as-needed basis. Fortunately, although the Lisfranc injury was diagnosed late, the patient recovered without significant deformity or loss of function. This case brings to light the importance of a systematic and thorough physical examination and evaluating the joints above and below the zone of injury.
